# A feasible molecular diagnostic strategy for rare genetic disorders within resource-constrained environments

**DOI:** 10.1007/s12687-023-00674-8

**Published:** 2023-10-10

**Authors:** Maria Mabyalwa Mudau, Heather Seymour, Patracia Nevondwe, Robyn Kerr, Careni Spencer, Candice Feben, Zané Lombard, Engela Honey, Amanda Krause, Nadia Carstens

**Affiliations:** 1 Division of Human Genetics, National Health Laboratory Service and School of Pathology, Faculty of Health Sciences, University of the Witwatersrand, Johannesburg, South Africa; 2 Division of Human Genetics, Faculty of Health Sciences, University of Cape Town, Cape Town, South Africa; 3 Department of Biochemistry, Genetics and Microbiology, Faculty of Natural and Agricultural Sciences, University of Pretoria, Pretoria, South Africa; 4 Genomics Platform, South African Medical Research Council, Cape Town, South Africa

**Keywords:** Custom-designed NGS targeted panel, Genetic testing, Rare diseases, Resource-constrained environment

## Abstract

Timely and accurate diagnosis of rare genetic disorders is critical, as it enables improved patient management and prognosis. In a resource-constrained environment such as the South African State healthcare system, the challenge is to design appropriate and cost-effective assays that will enable accurate genetic diagnostic services in patients of African ancestry across a broad disease spectrum. Next-generation sequencing (NGS) has transformed testing approaches for many Mendelian disorders, but this technology is still relatively new in our setting and requires cost-effective ways to implement. As a proof of concept, we describe a feasible diagnostic strategy for genetic disorders frequently seen in our genetics clinics (RASopathies, Cornelia de Lange syndrome, Treacher Collins syndrome, and CHARGE syndrome). The custom-designed targeted NGS gene panel enabled concurrent variant screening for these disorders. Samples were batched during sequencing and analyzed selectively based on the clinical phenotype. The strategy employed in the current study was cost-effective, with sequencing and analysis done at USD849.68 per sample and achieving an overall detection rate of 54.5%. The strategy employed is cost-effective as it allows batching of samples from patients with different diseases in a single run, an approach that can be utilized with rare and less frequently ordered molecular diagnostic tests. The subsequent selective analysis pipeline allowed for timeous reporting back of patients results. This is feasible with a reasonable yield and can be employed for the molecular diagnosis of a wide range of rare monogenic disorders in a resource-constrained environment.

## Introduction

There is limited genomic knowledge for most rare monogenic disorders in African patients as these populations are largely understudied (Baynam et al. 2020; Popejoy and Fullerton 2016). The paucity of genetic data stems from limited resources with regard to clinical genetic services but also limited genomic research, computational expertise, new/relevant technologies, and biomedical research infrastructure (Musanabaganwa et al. 2020). It is important for African rare disease research and clinical care infrastructure to be developed, as this will aid in the development of appropriate health systems to improve the diagnosis of genetic or rare disorders timeously and accurately on the continent. This benefits patients by enabling a better understanding of their prognosis, tailored management and surveillance, and more personalized treatment (Kamp et al. 2021). A precise genetic diagnosis also enables accurate genetic counselling for affected individuals and their relatives and offers options for testing at risk family members (Patch and Middleton 2018).

For rare monogenic disorders, it is challenging to develop diagnostic tests, as each one requires separate research, development, and validation which can be very costly (Di Resta et al. 2018). In addition, rare disorders have low sample volumes leading to few samples in a batch and/or long times between batches thereby increasing turnaround time. Thus, when developing a diagnostic test for rare disorders, it may be preferable to group them together in one sequencing run to save costs and time (Santani et al. 2017) especially in resource constrained laboratories.

Next-generation sequencing (NGS) technology represents a major breakthrough for molecular diagnostics and is rapidly replacing traditional variant screening approaches for monogenic disorders (Adams and Eng 2018). It is now possible to screen a large number of genes simultaneously through massively parallel sequencing, thereby significantly reducing both costs and time associated with variant screening (Xue et al. 2015; Yohe et al. 2015). However, financial and computational resources, as well as human expertise, are usually extremely limited for NGS-based genetic services in laboratories particularly within low-to-middle-income countries (LMIC) with constrained state healthcare systems (Baynam et al. 2020; Maxmen 2020; Musanabaganwa et al. 2020).

In South Africa, 80% of the population is served by the state healthcare system, but this system lacks sufficient resources to render this very important service, especially for diagnosis of genetic disorders. Limited genetic services (including medical genetics, genetic counselling, and basic diagnostic testing) are available but clustered mainly in four academic hospital complexes nationally, in Johannesburg, Cape Town (2), and Bloemfontein, in only three of nine provinces. For patients who do receive a diagnosis, this is at most a clinical diagnosis by a medical geneticist. There is unfortunately limited or no molecular diagnostic testing to confirm common clinical diagnoses, as such testing is frequently not available in the country (Kromberg et al. 2013). For most patients in other cities and, particularly, in more distant rural areas, even limited clinical and diagnostic genetic services are not available.

It is therefore vital to implement a testing strategy that is appropriate and effective in the South African (LMIC) setting. Data analysis for NGS is complex and requires high throughput computational software as well as bioinformatics expertise, and despite the decreasing costs of NGS technologies, these technologies remain expensive (Clark et al. 2018; Fujiki et al. 2018). Targeted NGS–based gene panel approaches remain cheaper to implement compared to whole genome or whole exome sequencing (WGS or WES) in diagnostic settings, and have consequently become a standard first tier test for most monogenic disorders (Beale et al. 2015; Clark et al. 2018; Fujiki et al. 2018; Marino et al. 2018).

For the successful implementation of NGS services for clinical diagnosis, a panel must be optimized for maximum use and a multidisciplinary team of scientists, researchers, and clinicians must work together to ensure and promote the success of phenotyping to genotyping to diagnosis of a genetic disorder (Lionel et al. 2018; Liu et al. 2019).

In the current study, a multi-disease NGS targeted gene panel was designed to balance cost, efficiency, turnaround time, data quality, and clinical utility. As a proof of concept, we describe a feasibility evaluation of a diagnostic strategy for a small group of rare genetic disorders. The current study focused on disorders representing more frequent diagnoses in genetic clinics, RASopathies, Cornelia de Lange syndrome, Treacher Collins syndrome, and CHARGE syndrome. These disorders represent some of the most common test requests at our facility as assessed by local medical geneticists.

## Methods

### Patient selection and ethics

Unrelated patients (*n* = 88) with four clinically distinct groups of disorders: RASopathies, Cornelia de Lange syndrome, Treacher Collins syndrome, and CHARGE syndrome, were selected to be included in the current study. The 88 samples in the current study did not include positive control, one patient who had prior testing in a private laboratory and a pathogenic variant identified (*LZTR1* variant) served as a sequencing control for targeted gene panel performance and data analysis pipeline.

Patients were selected based on the presence of suggestive clinical features associated with each of the phenotypes. Data was captured using a clinical tick-sheet, designed for the current study by the clinical team. The patients were identified and recruited through the genetic clinics of the Division of Human Genetics, National Health Laboratory Service and the University of the Witwatersrand, Johannesburg, and the Faculty of Health Sciences, University of Pretoria, Pretoria, Gauteng, South Africa, and consented for the research. This study was approved by the Human Research Ethics Committee (Medical) at the University of the Witwatersrand (M160830) and the University of Pretoria Research Ethics Committee (80/2018). Patient DNA was extracted from whole blood using the salting-out method (Miller et al. 1988), and DNA concentration and quality were assessed using the NanoDrop®ND-1000 spectrophotometer (NanoDrop Technologies, Wilmington, DE, USA) and Qubit fluorometer (Invitrogen by Thermo-Fisher Scientific, South Africa).

### Panel design

#### Gene selection

Selection of the genes to be included in the custom-designed targeted panel was informed by an extensive literature review and followed the ClinGen gene-disease clinical validity classification framework (Strande et al. 2017). Most genes were classified as being definitively associated with the phenotype, but some genes with preliminary evidence indicating possible association with the clinical phenotypes to be studied were also added. These additional genes were specifically added as the molecular epidemiology for the target disorders had not yet been characterized in African patients. The final list of genes included in the panel (Table 1) consisted of known genes associated with RASopathies (including Noonan syndrome 1 (OMIM#163950), Noonan syndrome with multiple lentigines (OMIM#151100), capillary malformation-arteriovenous malformation syndrome (OMIM#608354), Costello syndrome (OMIM#218040), Cardio-facio-cutaneous syndrome (OMIM#115150), Legius syndrome (OMIM#611431), and neurofibromatosis type 1 (OMIM#162200)), Cohesinopathies (Cornelia de Lange syndrome (OMIM#122470) and related phenotypes), and facial dysostoses (Treacher Collins syndrome (OMIM#154500), Nager syndrome (OMIM#154400), and Miller syndrome (OMIM#263750) and CHARGE syndrome (OMIM#214800)).

**Table 1:** Genes included in the custom-designed NGS targeted panel, their associated clinical phenotypes, and ClinGen classification at the time the panel was developed in August 2018

Clinical phenotype	51 genes (OMIM #)	ClinGen classification
RASopathies	*CBL* (165360), *LZTR1* (600574), *KRAS* (190070), *NRAS* (164790), *PTPN11* (176876), *RAF1* (164760), *SOS1* (182530), *RIT1* (609591), *SOS2* (601247), *RASA1* (139150), *BRAF* (164757), *MAP2K1* (176948), *MAP2K2* (601263), *SPRED1* (609291), *NF1* (613113), *NF2* (607379), *HRAS* (190020), *SHOC2* (602775)	Definitive association
Treacher Collins syndrome	*TCOF1* (606847), *POLR1C* (610060), *POLR1D* (613715)	Definitive association
CHARGE syndrome	*CHD7* (60889)	Definitive association
Cornelia de Lange syndrome	*NIPBL* (608667), *HDAC8* (300269), *RAD21* (606462), *SMC1A* (300040), *SMC3* (606062)	Definitive association
Additional preliminary and differential diagnosis genes	Facial dysostoses: *SF3B4* (605593), *DHODH* (126064), *EFTUD2* (603892), *SMAD4* (600993), *SMAD5* (603110), *SMAD6* (602931), *SMAD7* (602932), *EYA1* (601653), *PDS5A* (613200), *PDS5B* (605333), *SCC4* (614560), *STAG1* (604358), *ESCO2* (609353) Cornelia de Lange: *AFF4* (604417), *ANKRD11* (611192), *KMT2A* (159555), *TAF1* (313650), *TAF6* (602955), *SETD5* (615743), *SMARCB1* (601607), *ARID1B* (614556), RASopathies: *RRAS* (165090), *RASA2* (601589), *A2ML1* (610627)	Limited/Disputed association with the clinical phenotypes

#### Panel design tool

The Agilent SureDesign software (version 7.0.2.12) (Agilent technologies, CA, USA) was used to design the targeted panel, with a total probe region size of less than 499 kb. Only coding regions of these genes were included, with an additional 10 bases of flanking region added to cover exon–intron boundaries. The probe tiling parameters were edited to ensure full coverage of the genes with low complexity and repetitive regions.

### Library preparation and sequencing

Library preparation was done according to the Agilent SureSelect^QXT^ workflow, and approximately 25 ng of input DNA was required. DNA was fragmented and the fragments tagged with adaptors. Tagged fragments were then purified, and the desired size was selected using AMPure beads (Beckman Coulter, CA, USA). DNA quality and quantity of the amplicons were assessed using the Qubit fluorometer (Invitrogen by Thermo-Fisher Scientific, South Africa) and the Bioanalyser 2100 (Agilent technologies, CA, USA). The purified amplicons were hybridized to the custom designed capture library. Hybridized amplicons were captured on streptavidin-coated beads (Thermo Fisher Scientific Inc. MA, USA) then amplified using indexing primers. Indexed libraries were then purified and pooled together in equimolar amounts before sequencing. Pooled libraries (6–10 pM) were sequenced in low (8 samples) and higher throughout (24 samples) batches by utilizing the appropriate MiSeq reagent v2 kits (nano, micro, or standard kits) on the Illumina MiSeq Instrument following manufacturer protocols (Illumina, CA, USA).

### Variant calling and interpretation

Read alignment and variant calling were done using the Agilent SureCall data analysis software (version 4.0) using default parameters. The variant call file (VCF) of each sample was annotated using wANNOVAR (Chang and Wang 2012). Common variants were filtered out using > 1%, minor allele frequency (MAF) from available population databases (such as gnomAD global MAF, gnomAD MAF for African/African American, 1000 genomes global MAF, and 1000 genomes continental/African MAF), clinical significance, in-silico protein effect prediction tools, and mode of inheritance for each phenotype. Data analysis targeted only genes associated with the individual patient’s clinical phenotype.

Clinically significant variants were then classified using the American College of Medical Genetics and Genomics and Association for Molecular Pathology (ACMG-AMP) guidelines (Richards et al. 2015) to identify putative disease-causing variants. The data analysis pipeline employed in this study was limited to variants smaller than 50 bp. Integrative genomic viewer (IGV) was utilized to visualize the coverage depth of the target regions sequenced (Robinson et al. 2017). Sanger sequencing was used to verify the presence of putative disease-causing variants in patient samples.

### Costing analysis

This was done by assessing both the cost of testing done prior to this study in the patients where clinically significant variants were identified. This is to illustrate that resources were used on inappropriate but available testing. An estimation of how much testing would cost for these group of disorders if we were to sequence the relevant genes using Sanger sequencing was also done. This was informed by the costing model as well as the test price list of the National Health Laboratory Service (NHLS), South Africa. This costing is based on a service provision non-profit model. The NHLS provides diagnostic services to state patients in South Africa. In the current study, the price per sample was estimated using the reagents costs, equipment maintenance costs, and data analysis time.

## Results

### Panel design

One panel was developed for these disease groups to maximize resources and reduce costs because it allows for batching of different patient groups for testing on one panel. It is cheaper to use one panel rather than three panels for the same number of patients as there are fixed minimum costs on the custom panel depending on the probe regions covered. For example, using Agilent probe design tools, the probe region 1–499 kb falls under tier 1 pricing and this principle was used in the current study. If genes for three diseases are combined, the panel is used to maximum sequencing capacity. The gene panel targeted a total of 51 genes (Table 1).

### Panel performance

The probe region size of the custom NGS panel was 249.95 kb, containing 3375 probes in total, making the panel appropriately sized to work on smaller benchtop NGS systems. Our custom panel was used successfully to sequence on the Illumina MiSeq system using the nano, micro, and the standard MiSeq reagent v2 kits. The coverage obtained per target region ranged from 50 to 374 × . The panel was used to sequence patients with a clinical diagnosis or suspicion of RASopathies (*n* = 60), Treacher Collins syndrome (*n* = 9), CHARGE syndrome (*n* = 5), and Cornelia de Lange syndrome (*n* = 14). A total of 48 putative disease-causing variants (Class 4 or 5) were identified in 88 patients (Table 2), resulting in an overall diagnostic yield of 54.5%.

**Table 2:** Types of disease-causing variants identified per gene, association of gene variants with the disorder, and the detection rate per disorder

Genes with disease-causing variants	disease-causing-variants identified and type	Association to disease
RASopathy (33/60 = 55%)		
*PTPN11* = 10	Missense	Definitive
*NF1* = 7	5 = nonsense and 2 = frameshift	Definitive
*BRAF* = 7	Missense	Definitive
*LZTR1* = 3	Missense	Definitive
*SOS1* = 2	Missense	Definitive
*RAF1* = 2	Missense	Definitive
*MAP2K2* = 1	Missense	Definitive
*NRAS* = 1	Missense	Definitive
Treacher Collins (3/9 = 33%)		
*TCOF1* = 2	Frameshift	Definitive
*POLR1D* = 1	Frameshift	Definitive
Charge syndrome (4/5 = 80%)		
*CHD7* = 4	3 = nonsense and 1 = frameshift	Definitive
Cornelia de Lange (8/14 = 57%)		
*NIPBL* = 7	4 = frameshift, 1 = splice-site, and 2 missense	Definitive
*STAG1* = 1	1 nonsense	Preliminary

Of the disease causing variants identified in this current study, 70.8% (34) were previously reported and 29.2% (14) were novel and not reported in variant interpretation databases such as ClinVar, human genome mutation database HGMD®, or the scientific literature (Table 2).

The putative disease-causing variants identified using the custom panel in our current study were all confirmed using Sanger sequencing. Clinically significant variant (ClinVar ID: 2578395) was also identified in the *STAG1* gene, which was added as part of the differential diagnoses and indicating the importance of including the preliminary evidence genes in our panel.

The overall calculation of the diagnostic yield did not include the *LZTR1* variant that was used as a sequencing control. The *LZTR1*:c.2269C > T (p.Gln757Ter) was identified in a patient with features suggestive of Schwannomatosis. The sample was included as a control as the panel used had included the gene *LZTR1*.

### Cost-effectiveness

The cost effectiveness of the strategy in the current study is shown in Tables 3 and 4. Table 3 shows the testing that was done prior to the patients being enrolled in the current study, and Table 4 shows the estimation of the cost of unidirectional Sanger sequencing prices if we were to perform this on only the common genes associated with the four disease groups included in this study. Of the 48 patients with pathogenic/likely pathogenic variants, 32 had prior testing. This means that 66.67% had inefficient testing prior to this study leading to USD4801.55 of unnecessary spending.

**Table 3:** Cost of tests done prior to study recruitments

Testing done prior to enrolment in current study	Patients who had prior testing	Unit price per test in ZAR	Price in ZAR of patients who had prior testing	Price in USD of patients who had prior testing
MLPA for microdeletion/duplications syndromes	7	2693.10	18,851.70	1112.25
QF-PCR for aneuploidy testing	6	1967.86	11,807.16	696.62
Chromosomal analysis	9	1729.19	15,562.71	918.20
FISH testing for Di-George syndrome	3	1726.70	5180.10	305.63
Mucopolyssacharidosis enzyme testing	2	164.90	329.80	19.46
Cystic fibrosis testing	2	1833.00	3666.00	216.29
Array CGH	2	8492.38	16,984.76	1002.10
NF2 whole gene sequencing	1	9000.00	9000.00	531.00
Total	32		81,382.23	4801.55

Costing on Table 3 was done using the NHLS costing model. Exchange rate of ~ 17 ZAR (South African Rand) to USD (United States Dollar) at the time of the current study

**Table 4:** Price estimations of sequencing all gene regions/exons of common genes

Common genes per disorder	Number of exons	Estimated Sanger sequencing price (ZAR)	Estimated Sanger sequencing price (USD)
RASopathy			
*BRAF*	18	22,367.16	1321.20
*NF1*	61	75,799.82	4477.40
*PTPN11*	15	18,639.30	1101.00
*RAF1*	16	19,881.92	1174.40
*SOS1*	24	29,822.88	1761.60
Five common RASopathy genes	134	16,6511.08	9835.60
Treacher Collins			
*TCOF1*	26	32,308.12	1908.40
CHARGE syndrome			
*CHD7*	37	45,976.94	2715.8
Cornelia de Lange			
*NIPBL*	47	58,403.14	3449.8

Costing estimations on Table 4 was done using the NHLS costing model. Exchange rate of ~ 17 ZAR to USD at the time of the current study

Estimations of Sanger sequencing prices were performed for five genes most commonly associated with RASopathies (*BRAF*, *NF1*, *PTPN11*, *RAF1*, and *SOS1*), and three genes, *TCOF1*, *CHD7*, and *NIBPL* most commonly associated with Treacher Collins, CHARGE, and Cornelia de Lange syndromes, respectively. If we were to do unidirectional Sanger sequencing in all these genes at ~ ZAR1,242.62 (USD73.40) per region/exon, the estimated prices are shown in Table 4

It can be seen in Table 4 that if we were to perform Sanger sequencing on common genes only, it would be expensive and not feasible for our laboratory to perform testing on these genes. Using NGS targeted panel, each sample was sequenced at USD849.68 which targeted all the genes associated with the disorders as well as others considered part of the differential diagnosis and this included selective analysis based on the phenotype. This pricing includes laboratory staff labor, consumables, kits, and other servicing related to machine service and maintenance; the sequence pricing per sample is rapidly decreasing from when the study was conducted.

## Discussion

An urgent need exists for African genomic data that could be used to design and implement diagnostic strategies and treatments that are appropriate for patients of African ancestry (Maxmen 2020). The current study employed a principle of investigating known genes (associated with RASopathies, CHARGE syndrome, Treacher Collins and Cornelia de Lange syndrome) in a custom-designed targeted panel as a point of departure, achieving an overall diagnostic yield of 54.5%. Selective analysis of the relevant disorder associated genes included in the panel had a diagnostic yield of 55% for RASopathies, which was slightly lower than in a previous study (Čizmárová et al. 2016) where a RASopathy panel testing identified 68% clinically significant variants in a central European population. Most RASopathy genes such as *PTPN11*, *BRAF*, and *NF1* reported to be associated with 50% Noonan syndrome, 75% Cardio-facio-cutaneous syndrome, and > 95% neurofibromatosis type 1 cases, respectively (Abdel-Aziz et al. 2021; Athota et al. 2020; Başaran 2021; Pierpont et al. 2014), and yielded expected detection rates as reported in the literature. Unexpected finding was observed in *SOS1* and *RAF1* genes which are both reported to be associated with 10–20% of Noonan syndrome cases (Alkaya et al. 2021), and were observed at lower detection rate of 6% each in the current study. However, each gene had two variants identified which is relatively low to make significant comparisons. CHARGE syndrome patients had a diagnostic yield of 80%, similar to the 80% reported by Janssen et al. in a Caucasian cohort using whole exome sequencing (Janssen et al. 2012; Ravenswaaij-Arts and Martin 2017). The diagnostic yield of 33% obtained for Treacher Collins syndrome patients was lower than the 71.4% of Fan et al. (2019) reported when using WES and Sanger sequencing. For Cornelia de Lange patients, 57% detection rate was achieved in the current study, lower compared to Braunholz et al. (2015) who achieved 70% detection rate while screening Cornelia de Lange patients using NGS targeted gene panel. The different yields of known genes could be reflective of differences in the African genome (Choudhury et al. 2020). They could also indicate inclusion of patients with alternative diagnoses. Further studies will assist in clarifying these explanations.

The detection rates achieved per disorders studied in the current study are comparable to the literature but are lower in some cases. This likely reflects diversity of variants in an African cohort or the small sample size. This could also be attributed to most syndromes not being well described in African patients which sometimes makes recognizing some features in African patients difficult (Caelers 2023; Omotoso et al. 2022). Identification of clinically significant variants in well-described genes could serve as a first line investigation and indicates the great potential this panel has for achieving a diagnosis in a high percentage of patients. Importantly, one variant was identified in a preliminary evidence and differential diagnosis gene, indicating the importance of including genes associated with phenotypically overlapping syndromes when designing targeted gene panel for diagnostic testing. New/novel variants (29%) identified in this study provides new insights into African clinically significant variants profiles in some more common genetic syndromes that overlap but differ from those in the published literature, and may, in some cases, require local tailored diagnostic approaches (Zhong et al. 2021). Panels could also be improved over time, as relevant research demonstrates the need for inclusion of genes.

The strategy presented in the current study is cost-effective and could be adopted for establishing diagnostic testing for monogenic disorders in a resource-constrained environment. It is valuable to confirm a diagnosis of a genetic disease as this enables proper management, treatment, and other important interventions necessary to improve the health of the patient (Alliance and Health 2010; Marian 2020). Before NGS was introduced, no molecular confirmatory testing was available in South Africa for this group of disorders due to their heterogeneous nature and the limitations of Sanger sequencing. Prior to this study, patients received non-optimal and costly testing that did not identify their pathogenic cause. It also illustrates that clinicians use what is available, even if non-ideal, testing to try to reach a diagnosis, thus incurring significant cost. As illustrated in Table 4, it can be seen that using NGS, each patient was sequenced at USD849.68 and this covered all the genes included in the targeted panel associated with the four groups of disorders including their differential diagnosis compared to USD9, 835.60 which was estimated as the possible cost if Sanger sequencing was performed for RASopathy patients only for example. This illustrates the value of NGS as a molecular diagnostic tool because it enables parallel testing of many genes for less than the cost of Sanger sequencing one gene on (Hu et al. 2021).

There is an increased interest in using the targeted NGS gene panel approach for variant screening of genetic diseases with relatively limited locus heterogeneity (Castellanos et al. 2017). Targeted panels are typically designed for a group of related genes, disorders, or phenotypes (Gulilat et al. 2019; Santani et al. 2017). Targeted gene panels are still considerably cheaper than other NGS-based methods in LMIC, although the prices may seem high compared to the northern hemisphere costs. This may be because most of the NGS reagents are shipped from the northern hemisphere, through third party distributors as there are no local manufacturers of most of the reagents used in the NGS testing and this may increase costs as they add margins. Low volumes, limited competition, sole suppliers, and high import duties also contribute to cost.

The approach employed in the current study differs from conventional panel designs as we grouped clinically unrelated disorders in one targeted panel based on the reported need in our genetic clinics to try to achieve batching and costing efficiency. For maximum efficiency, as many genes as possible were included on a panel (based on the sequencing capacity of the lowest panel pricing option) while still allowing for adequate coverage. Selective analysis allowed us to limit analysis and report only on the genes relevant to each patient’s clinical phenotype. This helped to streamline the analysis step, and this process has allowed us to reach a clinically acceptable turn-around time which ranged from 4 weeks (patients with no variants identified) to 16 weeks; this included time for validation of the variants using Sanger sequencing.

The putative disease-causing variants identified using the custom panel in our current study were confirmed using Sanger sequencing. This was done in order to validate the results before reporting these back to the patients, as this was the first NGS study in our laboratory. The quality of the reads obtained using the targeted panel was high, all had > Q30, and the sequencing coverage depth was above 30 × for all the samples, as visualized on the integrative genome viewer (IGV). This validation together with fine tuning of parameters used for NGS data quality control procedure has enabled us to determine when to perform Sanger validation as described in various guidelines for NGS implementation in a diagnostic setting (Aziz et al. 2015; Hume et al. 2019; Matthijs et al. 2016) and to manage cost further. In our current setting, Sanger sequencing will be used where variant calling is uncertain, such as from homopolymer regions or poorly covered regions of the genome and for cascade testing in families (Baudhuin et al. 2015; Beck et al. 2016; Mu et al. 2016).

All validated disease-causing variants were reported back to the patients; this gave us the opportunity to improve the decision-making on the management of the patient’s disorder. We were also able to provide informed genetic counselling services to the patients. Although the panel was validated on Treacher Collins syndrome, CHARGE syndrome, Cornelia de Lange syndrome, and RASopathy syndromes, this approach of combining conditions to maximize sequencing capacity and analyzing many rare conditions simultaneously on one test can be extended to other genetic disorders. This panel was optimized and validated on the Illumina MiSeq platform, but the approach is amenable to smaller bench-top NGS platforms such as the Illumina iSeq, which requires smaller capital investment, as well as other NGS chemistries. As many diagnostic laboratories move towards using NGS in low resource environments, cost-effectiveness should be a high priority due to the number of competing demands in the healthcare sector. There are factors that influence costs, and these should be considered when developing NGS based diagnostic tests. Apart from reagents and instrument costs, there are human resource costs, particularly the time of scientists involved in the analysis, data storage costs and logistics, validations of procedures, and performing additional confirmatory procedures (Kingsmore et al. 2012; Radomski et al. 2020). Although it is acknowledged that available NGS services may be cheaper in large laboratories in the USA or Europe, these services can only be offered to South African patients that can afford to pay for private genetic testing service. In our setting, there are patients who are referred to laboratories abroad for various genetic tests that are not currently offered; however, this is often based on affordability. The current study aimed to present a feasible way to offer this service in a low resourced environment like ours particularly to patients who rely on the government health system for care. In addition to being able to offer a cost-effective service, this allows LMIC laboratories to perform relevant research and acquire competence in these scarce skills.

## Conclusion

The current study demonstrated the feasibility and cost-effectiveness of combining clinically distinct diseases on one sequencing panel, followed by selective analysis as a strategic diagnostic tool for a group of rare genetically heterogeneous diseases in a limited-resourced setting. This strategy has the potential to improve diagnostic service as it allows batching of different tests in one run utilizing the sequencing capacity to minimize costs and reduce turnaround time. The knowledge gained from this study serves as a foundation to develop a more appropriate and cost-effective diagnostic testing strategy for monogenic disorders in a laboratory with limited resources thereby providing potential for increasingly effective genetic diagnostic testing to the South African public health care system. Similar value would be expected in other limited resource environments.

## Data Availability

The datasets used or analyzed during the current study are available on reasonable request. Variant information was submitted to ClinVar and can be viewed under Organization ID 508172.

## Declarations

### Disclaimer

The views and opinions expressed and conclusions arrived at are those of the authors and are not necessarily to be attributed to the NRF, the SAMRC, or the NHLS.

## References

[CR1] Abdel-Aziz NN, El-Kamah GY, Khairat RA, Mohamed HR, Gad YZ, El-Ghor AM, Amr K (2021). Mutational spectrum of NF1 gene in 24 unrelated Egyptian families with neurofibromatosis type 1. Mol Genet Genomic Med.

[CR2] Adams DR, Eng CM (2018). Next-generation sequencing to diagnose suspected genetic disorders. N Engl J Med.

[CR3] Alkaya DU, Lissewski C, Yeşil G, Zenker M, Tüysüz B (2021). Expanding the clinical phenotype of RASopathies in 38 Turkish patients, including the rare LZTR1, RAF1, RIT1 variants, and large deletion in NF1. Am J Med Genet A.

[CR4] Alliance G, Health, D. of C.D. of (2010) Diagnosis of a genetic disease, understanding genetics: a District of Columbia guide for patients and health professionals. Genetic Alliance. 23586106

[CR5] Athota JP, Bhat M, Nampoothiri S, Gowrishankar K, Narayanachar SG, Puttamallesh V, Farooque MO, Shetty S (2020). Molecular and clinical studies in 107 Noonan syndrome affected individuals with PTPN11 mutations. BMC Med Genet.

[CR6] Aziz N, Zhao Q, Bry L, Driscoll DK, Funke B, Gibson JS, Grody WW, Hegde MR, Hoeltge GA, Leonard DGB, Merker JD, Nagarajan R, Palicki LA, Robetorye RS, Schrijver I, Weck KE, Voelkerding KV (2015). College of American pathologists’ laboratory standards for next-generation sequencing clinical tests. Arch Pathol Lab Med.

[CR7] Başaran S (2021) The effectiveness of PTPN11 gene analysis in the prenatal diagnosis of noonan syndrome. J Istanbul Fac Med 84:34–39. 10.26650/IUITFD.2020.803356

[CR8] Baudhuin LM, Lagerstedt SA, Klee EW, Fadra N, Oglesbee D, Ferber MJ (2015). Confirming variants in next-generation sequencing panel testing by Sanger sequencing. J Mol Diagn.

[CR9] Baynam GS, Groft S, van der Westhuizen FH, Gassman SD, du Plessis K, Coles EP, Selebatso E, Selebatso M, Gaobinelwe B, Selebatso T, Joel D, Llera VA, Vorster BC, Wuebbels B, Djoudalbaye B, Austin CP, Kumuthini J, Forman J, Kaufmann P, Chipeta J, Gavhed D, Larsson A, Stojiljkovic M, Nordgren A, Roldan EJA, Taruscio D, Wong-Rieger D, Nowak K, Bilkey GA, Easteal S, Bowdin S, Reichardt JKV, Beltran S, Kosaki K, van Karnebeek CDM, Gong M, Shuyang Z, Mehrian-Shai R, Adams DR, Puri RD, Zhang F, Pachter N, Muenke M, Nellaker C, Gahl WA, Cederroth H, Broley S, Schoonen M, Boycott KM, Posada M (2020). A call for global action for rare diseases in Africa. Nat Genet.

[CR10] Beale S, Sanderson D, Sanniti A, Dundar Y, Boland A (2015) Economics, A scoping study to explore the cost-effectiveness of next-generation sequencing compared with traditional genetic testing for the diagnosis of learning disabilities in children. NIHR Journals Library 10.3310/hta19460 PMC4967812 26132578

[CR11] Beck TF, Mullikin JC, NISC comparative sequencing program, Biesecker LG (2016) Systematic Evaluation of Sanger Validation of Next-Generation Sequencing Variants. Clin Chem 62:647–654. 10.1373/clinchem.2015.249623 10.1373/clinchem.2015.249623 PMC4878677 26847218

[CR12] Braunholz D, Obieglo C, Parenti I, Pozojevic J, Eckhold J, Reiz B, Braenne I, Wendt KS, Watrin E, Vodopiutz J, Rieder H, Gillessen-Kaesbach G, Kaiser FJ (2015). Hidden mutations in Cornelia de Lange syndrome limitations of sanger sequencing in molecular diagnostics. Hum Mutat.

[CR13] Caelers D (2023). African genomic data deficit threatens global human health. Nature Africa.

[CR14] Castellanos E, Gel B, Rosas I, Tornero E, Santín S, Pluvinet R, Velasco J, Sumoy L, del Valle J, Perucho M, Blanco I, Navarro M, Brunet J, Pineda M, Feliubadaló L, Capellá G, Lázaro C, Serra E (2017). A comprehensive custom panel design for routine hereditary cancer testing: preserving control, improving diagnostics and revealing a complex variation landscape. Sci Rep.

[CR15] Chang X, Wang K (2012). wANNOVAR: annotating genetic variants for personal genomes via the web. J Med Genet.

[CR16] Choudhury A, Aron S, Botigué LR, Sengupta D, Botha G, Bensellak T, Wells G, Kumuthini J, Shriner D, Fakim YJ, Ghoorah AW, Dareng E, Odia T, Falola O, Adebiyi E, Hazelhurst S, Mazandu G, Nyangiri OA, Mbiyavanga M, Benkahla A, Kassim SK, Mulder N, Adebamowo SN, Chimusa ER, Muzny D, Metcalf G, Gibbs RA, Rotimi C, Ramsay M, Adeyemo AA, Lombard Z, Hanchard NA (2020). High-depth African genomes inform human migration and health. Nature.

[CR17] Čizmárová M, Hlinková K, Bertok S, Kotnik P, Duba HC, Bertalan R, Poločková K, Košťálová Ľ, Pribilincová Z, Hlavatá A, Kovács L, Ilenčíková D (2016). New mutations associated with rasopathies in a central European population and genotype-phenotype correlations. Ann Hum Genet.

[CR18] Clark MM, Stark Z, Farnaes L, Tan TY, White SM, Dimmock D, Kingsmore SF (2018). Meta-analysis of the diagnostic and clinical utility of genome and exome sequencing and chromosomal microarray in children with suspected genetic diseases. NPJ Genom Med.

[CR19] Di Resta C, Galbiati S, Carrera P, Ferrari M (2018). Next-generation sequencing approach for the diagnosis of human diseases: open challenges and new opportunities. EJIFCC.

[CR20] Fan X, Wang Y, Fan Y, Du H, Luo N, Zhang S, Chen X (2019). TCOF1 pathogenic variants identified by whole-exome sequencing in Chinese Treacher Collins syndrome families and hearing rehabilitation effect. Orphanet J Rare Dis.

[CR21] Fujiki R, Ikeda M, Yoshida A, Akiko M, Yao Y, Nishimura M, Matsushita K, Ichikawa T, Tanaka T, Morisaki H, Morisaki T, Ohara O (2018). Assessing the accuracy of variant detection in cost-effective gene panel testing by next-generation sequencing. J Mol Diagn.

[CR22] Gulilat M, Lamb T, Teft WA, Wang J, Dron JS, Robinson JF, Tirona RG, Hegele RA, Kim RB, Schwarz UI (2019). Targeted next generation sequencing as a tool for precision medicine. BMC Med Genomics.

[CR23] Hu T, Chitnis N, Monos D, Dinh A (2021) Next-generation sequencing technologies: an overview. Human Immunology, Next Generation Sequencing and its Application to Medical Laboratory Immunology 82:801–811. 10.1016/j.humimm.2021.02.012 10.1016/j.humimm.2021.02.012 33745759

[CR24] Hume S, Nelson TN, Speevak M, McCready E, Agatep R, Feilotter H, Parboosingh J, Stavropoulos DJ, Taylor S, Stockley TL (2019). CCMG practice guideline: laboratory guidelines for next-generation sequencing. J Med Genet.

[CR25] Janssen N, Bergman JEH, Swertz MA, Tranebjaerg L, Lodahl M, Schoots J, Hofstra RMW, van Ravenswaaij-Arts CMA, Hoefsloot LH (2012). Mutation update on the CHD7 gene involved in CHARGE syndrome. Hum Mutat.

[CR26] Kamp M, Krause A, Ramsay M (2021). Has translational genomics come of age in Africa?. Hum Mol Genet.

[CR27] Kingsmore SF, Lantos JD, Dinwiddie DL, Miller NA, Soden SE, Farrow EG, Saunders CJ (2012). Next-generation community genetics for low- and middle-income countries. Genome Med.

[CR28] Kromberg JGR, Sizer EB, Christianson AL (2013). Genetic services and testing in South Africa. J Community Genet.

[CR29] Lionel AC, Costain G, Monfared N, Walker S, Reuter MS, Hosseini SM, Thiruvahindrapuram B, Merico D, Jobling R, Nalpathamkalam T, Pellecchia G, Sung WWL, Wang Z, Bikangaga P, Boelman C, Carter MT, Cordeiro D, Cytrynbaum C, Dell SD, Dhir P, Dowling JJ, Heon E, Hewson S, Hiraki L, Inbar-Feigenberg M, Klatt R, Kronick J, Laxer RM, Licht C, MacDonald H, Mercimek-Andrews S, Mendoza-Londono R, Piscione T, Schneider R, Schulze A, Silverman E, Siriwardena K, Snead OC, Sondheimer N, Sutherland J, Vincent A, Wasserman JD, Weksberg R, Shuman C, Carew C, Szego MJ, Hayeems RZ, Basran R, Stavropoulos DJ, Ray PN, Bowdin S, Meyn MS, Cohn RD, Scherer SW, Marshall CR (2018). Improved diagnostic yield compared with targeted gene sequencing panels suggests a role for whole-genome sequencing as a first-tier genetic test. Genet Med.

[CR30] Liu Z, Zhu L, Roberts R, Tong W (2019). Toward clinical implementation of next-generation sequencing-based genetic testing in rare diseases: where are we?. Trends Genet.

[CR31] Marian AJ (2020). Clinical Interpretation and Management of Genetic Variants. JACC: Basic Transl Sci.

[CR32] Marino P, Touzani R, Perrier L, Rouleau E, Kossi DS, Zhaomin Z, Charrier N, Goardon N, Preudhomme C, Durand-Zaleski I, Borget I, Baffert S (2018). Cost of cancer diagnosis using next-generation sequencing targeted gene panels in routine practice: a nationwide French study. Eur J Hum Genet.

[CR33] Matthijs G, Souche E, Alders M, Corveleyn A, Eck S, Feenstra I, Race V, Sistermans E, Sturm M, Weiss M, Yntema H, Bakker E, Scheffer H, Bauer P (2016). Guidelines for diagnostic next-generation sequencing. Eur J Hum Genet.

[CR34] Maxmen A (2020). The next chapter for African genomics. Nature.

[CR35] Miller SA, Dykes DD, Polesky HF (1988). A simple salting out procedure for extracting DNA from human nucleated cells. Nucleic Acids Res.

[CR36] Mu W, Lu H-M, Chen J, Li S, Elliott AM (2016). Sanger confirmation is required to achieve optimal sensitivity and specificity in next-generation sequencing panel testing. J Mol Diagn.

[CR37] Musanabaganwa C, Mihigo B, Tumusime R, Uwanyirigira M, da Rocha J, Hayat M, Govender M, Buto P, Nyunga T, Ramesar RS, Rotimi C, Souopgui J, Wonkam A, Williams SM, Jansen S, Ramsay M, Mutesa L (2020). Building skills and resources for genomics, epigenetics, and bioinformatics research for Africa: report of the Joint 11th Conference of the African Society of Human Genetics and 12th H3Africa Consortium, 2018. Am J Trop Med Hyg.

[CR38] Omotoso OE, Teibo JO, Atiba FA, Oladimeji T, Adebesin AO, Babalghith AO (2022). Bridging the genomic data gap in Africa: implications for global disease burdens. Glob Health.

[CR39] Patch C, Middleton A (2018). Genetic counselling in the era of genomic medicine. Br Med Bull.

[CR40] Pierpont MEM, Magoulas PL, Adi S, Kavamura MI, Neri G, Noonan J, Pierpont EI, Reinker K, Roberts AE, Shankar S, Sullivan J, Wolford M, Conger B, Santa Cruz M, Rauen KA (2014). Cardio-facio-cutaneous syndrome: clinical features, diagnosis, and management guidelines. Pediatrics.

[CR41] Popejoy AB, Fullerton SM (2016). Genomics is failing on diversity. Nature News.

[CR42] Radomski TR, Feldman R, Huang Y, Sileanu FE, Thorpe CT, Thorpe JM, Fine MJ, Gellad WF (2020). Evaluation of low-value diagnostic testing for 4 common conditions in the Veterans Health Administration. JAMA Netw Open.

[CR43] Richards S, Aziz N, Bale S, Bick D, Das S, Gastier-Foster J, Grody WW, Hegde M, Lyon E, Spector E, Voelkerding K, Rehm HL, Laboratory Quality Assurance Committee ACMG (2015). Standards and guidelines for the interpretation of sequence variants: a joint consensus recommendation of the American College of Medical Genetics and Genomics and the Association for Molecular Pathology. Genet Med.

[CR44] Robinson JT, Thorvaldsdóttir H, Wenger AM, Zehir A, Mesirov JP (2017). Variant review with the integrative genomics Viewer. Cancer Res.

[CR45] Santani A, Murrell J, Funke B, Yu Z, Hegde M, Mao R, Ferreira-Gonzalez A, Voelkerding KV, Weck KE (2017). Development and validation of targeted next-generation sequencing panels for detection of germline variants in inherited diseases. Arch Pathol Lab Med.

[CR46] Strande NT, Riggs ER, Buchanan AH, Ceyhan-Birsoy O, DiStefano M, Dwight SS, Goldstein J, Ghosh R, Seifert BA, Sneddon TP, Wright MW, Milko LV, Cherry JM, Giovanni MA, Murray MF, O’Daniel JM, Ramos EM, Santani AB, Scott AF, Plon SE, Rehm HL, Martin CL, Berg JS (2017). Evaluating the clinical validity of gene-disease associations: an evidence-based framework developed by the clinical genome resource. Am J Hum Genet.

[CR47] van Ravenswaaij-Arts C, Martin DM (2017). New insights and advances in CHARGE syndrome: diagnosis, etiologies, treatments, and research discoveries. Am J Med Genet C Semin Med Genet.

[CR48] Xue Y, Ankala A, Wilcox WR, Hegde MR (2015). Solving the molecular diagnostic testing conundrum for Mendelian disorders in the era of next-generation sequencing: single-gene, gene panel, or exome/genome sequencing. Genet Med.

[CR49] Yohe S, Hauge A, Bunjer K, Kemmer T, Bower M, Schomaker M, Onsongo G, Wilson J, Erdmann J, Zhou Y, Deshpande A, Spears MD, Beckman K, Silverstein KAT, Thyagarajan B (2015). Clinical validation of targeted next-generation sequencing for inherited disorders. Arch Pathol Lab Med.

[CR50] Zhong Y, Xu F, Wu J, Schubert J, Li MM (2021). Application of next generation sequencing in laboratory medicine. Ann Lab Med.

